# Reliability and Validity of the Korean Version of Lung Cancer Screening Health Belief Scale

**DOI:** 10.3390/healthcare11111525

**Published:** 2023-05-23

**Authors:** Mi-Kyoung Cho, Yoon-Hee Cho

**Affiliations:** 1 Department of Nursing Science, Chungbuk National University, Cheongju 28644, Republic of Korea; ciamkcho@gmail.com; 2 Department of Nursing, College of Nursing, Dankook University, Cheonan 31116, Republic of Korea

**Keywords:** LCSHBS, validity, reliability, health belief, lung cancer, early detection of cancer

## Abstract

The purpose of this study was to verify the validity and reliability of the LCSHBS-K. This was a methodological study. The participants were adults aged between 50 and 74 years old, according to the selection criteria for lung cancer screening presented by the Comprehensive Cancer Network clinical practice guidelines in oncology recommendations. This study included 204 high-risk individuals who had not been diagnosed with lung cancer. The collected data were analyzed using the IBM SPSS Statistics software 26.0 version (IBM, New York, NY, USA). The reliability was analyzed by Cronbach’s α for internal consistency, and the concurrent validity was analyzed by Pearson’s correlation coefficients to identify the correlations with the health belief scale for Korean adults. To test the convergent validity, the average variance extracted (AVE) and composite reliability (CR) were calculated using confirmatory factor analysis. In addition, the model fit for a tool was CMIN (χ^2^/df), SRMR, RMSEA, GFI, and CFI as a comparative fit index. The discriminant validity was tested based on AVE > r^2^. The average age of the participants was 55.49 (SD = 5.07), the average smoking history was 29.55 (SD = 8.12) years, and the average number of cigarettes smoked per day was 12.18 (SD = 7.77). The goodness of fit met the criteria with GFI = 0.81 (criteria > 0.9), CMIN = 1.69 (criteria < 2), SRMR = 0.06 (criteria < 0.08), RMSEA = 0.058 (criteria < 0.06), and CFI = 0.91 (criteria > 0.9). The LCSHBS-K showed a statistically significant positive correlation with the HBS (r = 0.32 (*p* < 0.001)). Cronbach’s α was 0.80 for all the items in the LCSHBS-K. Therefore, the validity and reliability of the LCSHBS-K tool were confirmed. Based on the results of this study, the Korean version of the LCSHBS tool was found to be suitable for screening lung cancer in high-risk groups in Korea.

## 1. Introduction

Cancer is one of the most common causes of death worldwide, and lung cancer is one of the most fatal types of cancer, irrespective of race or sex [1]. The 2021 statistics on the causes of death in South Korea showed that cancer was the number one cause of death in both men and women aged 40 years or above, with lung cancer causing the highest level of mortality in both sexes. The death rate due to lung cancer was 54.0 deaths per 100,000 men and 18.8 deaths per 100,000 women, indicating a continuous increase in mortality compared to previous years [2]. The highest mortality rate was observed in the population older than 60 years [2].

A high percentage of cases of lung cancer are diagnosed when the disease has already progressed. The localized lung cancer diagnosis rate in patients diagnosed between 2012 and 2016 was only 21.0%, and the 5-year relative survival rate was 28.2%, which is relatively lower than that of other cancer types [3]. Nevertheless, early detection of lung cancer increases the 5-year survival rate to 60–70% [4]. Therefore, the diagnosis of asymptomatic lung cancer using an effective screening method is important for timely treatment and the extension of life.

Smoking is associated with all types of lung cancer (approximately 90%) and is the most significant risk factor. A large-scale comparative clinical study conducted in 2011 in the U.S. screened for lung cancer using low-dose chest computed tomography (CT) in individuals aged 55–74 years at high risk for lung cancer with a history of smoking of 30 pack-years. The study reported a 20% lower lung cancer mortality in the group that was screened for lung cancer as compared to the control group, and an overall decrease in mortality of 7% [5]. Low-dose chest CT is a sensitive tool for the early detection of lung cancer [6]. Thus, the U.S. Preventive Services Task Force and the South Korean government recommend chest CT for screening high-risk groups [5,7]. However, screening using low-dose chest CT is yet to be widely accepted worldwide, despite its advantages related to morbidity and mortality [4].

Previous studies have reported the factors associated with a low rate of participation in lung cancer screening programs, including psychological and cognitive variables (social stigma, distrust, fatalism, anxiety, fear, and a low level of knowledge of lung cancer and lung cancer screening), health beliefs (perceived risk, perceived benefit, perceived barrier, and self-efficacy), medical experts’ recommendations, media exposure, and others [8,9]. The concept of health belief was first suggested by a social psychologist in the U.S. Public Health Service in the early 1950s to account for the low rate of participation in programs for disease prevention and health screening tests [10]. Health belief is a critical predictor of various health behaviors, such as smoking, exercise, patient roles, and the use of healthcare services, and accounts for preventive health behaviors related to specific diseases [11].

The expanded Health Belief Model (EHBM) is a commonly used model to describe participation in cancer screening, such as lung cancer [9]. The EHBM includes the perceived risk, perceived benefits, perceived barriers, self-efficacy, and perceived severity [9,11]. The EHBM states that the probability of an individual participating in a lung cancer screening test increases as the levels of perceived benefit, perceived barriers, and self-efficacy increase, supporting the structure of the conceptual model describing the participation of high-risk groups in lung cancer screening [4]. Hence, assessing and understanding personal beliefs regarding lung cancer screening are critical for improving the rate of screening for lung cancer in high-risk groups. Thus, the Lung Cancer Screening Health Belief Scale (LCSHBS) was developed in English to measure the beliefs of individuals regarding lung cancer screening [12]. The LSCHBS includes health beliefs about lung cancer screening: (1) perceived risk of lung cancer; (2) perceived benefits of lung cancer screening; (3) perceived barriers to lung cancer screening; and (4) self-efficacy for lung cancer screening [9]. Perceived severity, which was included in the EHBM, was excluded because it has been reported that it is not useful for cancer screening, which is already recognized as a serious disease [9]. The validity and reliability of the LSCHBS have been tested and confirmed. However, applying the tool in South Korea requires the verification of the tool’s validity and reliability in high-risk patients in Korea to allow the collection of stronger evidence.

This study aimed to produce and validate a Korean version of the LCSHBS originally developed by Carter-Harris et al. [12] by translating the tool according to the current statuses in South Korea among Korean adults at high risk for lung cancer.

## 2. Materials and Methods

### 2.1. Study Design

This methodological study translated the LCSHBS developed by Carter-Harris et al. [12] and verified its validity and reliability in Korean adults at high risk for lung cancer.

### 2.2. Participants

For the participants in this study, the inclusion criteria were based on those for lung cancer screening suggested by the National Comprehensive Cancer Network Lung Cancer Screening Version 3. 2018 Clinical Practice Guidelines in Oncology Recommendation [13], as follows: adults aged ≥50 and <74 years, with a history of smoking 20 packs/year in the past 15 years, who belonged to a high-risk group, had not been diagnosed with lung cancer, and were able to use their smartphone or PC independently to visit the survey URL and voluntarily complete the online consent and questionnaire. The exclusion criteria were adults aged <50 years with a history of lung cancer diagnosis or surgery for lung cancer; those who used a mobile phone that did not allow the user to access the survey URL set by the researchers; those who could not read, comprehend, give consent, or respond to the questionnaire; and those who either did not agree to participate, or dropped out in the middle of the survey. The sample size was estimated based on the criteria of 200 individuals in a confirmatory factor analysis and the claim that n ≥ 200 as the sample size for a factor analysis is moderately adequate; hence, the data were collected from 204 individuals satisfying the inclusion and exclusion criteria [14,15,16].

### 2.3. Research Ethics

After receiving approval from the institutional review board of the educational institution to which the researchers belong, data were collected using a recruitment ad on the panels of an online survey provider. The purpose, procedures, and data collection methods were presented on the first page of the online survey site. The website also clearly stated that participation was entirely voluntary, and the participants had the right to discontinue taking the survey or refuse to respond to the questionnaire. The page also included the contact details of the researchers and explained that no data would be included in the analysis if the participant dropped out of the survey, and that there would be no disadvantage caused by withdrawal from or rejection of the survey.

### 2.4. Tools

#### 2.4.1. Participant Characteristics

The characteristics of the participants in this study that were recorded included age, sex, education, marital status, number of family members living together, religion, residence, economic status, perceived stress, health status, participation in the screening test (CT), history of pulmonary disease, cigarette smoking period, amount of cigarette smoking, e-cigarette (cigarette and liquid type) smoking, e-cigarette smoking period, and level of e-cigarette smoking.

#### 2.4.2. Lung Cancer Screening Health Belief Scales (LCSHBS)

The Lung Cancer Screening Health Belief Scales (LCSHBS) consist of four dimensions: perceived risk, perceived benefit, perceived barrier, and self-efficacy. The definition of each dimension is as follows: perceived risk in relation to lung cancer screening is the belief of an individual in the probability of his or her acquiring lung cancer; the perceived benefit is the belief of an individual in the positive outcomes of lung cancer screening resulting from adhering to the recommendations to reduce the risk of lung cancer; the perceived barrier is the participation and cost-related challenges faced by an individual in relation to lung cancer screening; self-efficacy is the self-confidence in being able to perform every task involved in lung cancer screening [12]. The LCSHBS in this study was originally developed by Carter-Harris et al. [12] and included three questions on the perceived risk of lung cancer, six questions on the perceived benefit, 17 questions on the perceived barrier, and nine questions on self-efficacy related to lung cancer screening, which amounted to a total of 35 questions. Each question was rated on a 4-point Likert scale ranging from 1 (‘strongly disagree’) to 4 (‘strongly agree’). Higher scores indicated a stronger belief in each dimension of the LCSHBS. The Cronbach’s α ranged from 0.80 to 0.92 at the time of tool development [12], 0.78 to 0.93 in this study.

#### 2.4.3. Health Beliefs Scale (HBS)

Health belief is an individual’s subjective belief that underlies his or her behavior toward disease prevention. The dimensions of the health belief scale include perceived sensitivity, perceived severity, perceived benefit, perceived barrier, and motivation to health. The HBS used in this study was developed by Moon [17] for adults and includes eight questions on perceived sensitivity, eight questions on perceived benefit, nine questions on perceived severity, nine questions on the perceived barrier, nine questions on motivation to health, and 12 questions on self-efficacy. The HBS by Moon was developed in Korean. Since the HBS was developed, it has been used to measure not only health beliefs of healthy people, but also health beliefs for health promotion behaviors of cancer patients, such as gastric cancer in Korea. Each of the 55 questions was rated on a 4-point Likert scale from 1 (‘strongly disagree’) to 4 (‘strongly agree’), with a score range of 55–220. Higher scores indicated higher levels of health beliefs. Cronbach’s α was 0.89 (dimension: 0.67–0.80) at the time of tool development by Moon [17] and 0.87 (dimension: 0.66–0.72) in the present study.

### 2.5. Study Procedure

Written approval was obtained from the tool developer Carter-Harris to translate and produce a Korean version of the original English version of the LCSHBS (LCSHBS-K) and verify its validity and reliability. This study complied with the COSMIN checklist [18], a tool development guideline. This study applied the Brislin method [19] for back translation to effectively enhance cross-cultural validity. The original English version of the LCSHBS was translated into Korean by a bilingual expert. A panel of experts (two professors of nursing, one nurse specialist in lung cancer, and one internal medicine specialist) determined the cross-cultural equivalence and lexical errors of the translated Korean questions. The clarity of each question was also confirmed. The questions that were revised by the experts were then translated back to English by a different bilingual expert who had not read the original English questions. The English and Korean versions of each question were compared, and the translated questions were revised until the two versions were equivalent. In both the forward and back translation processes, the focus was on conceptual equivalence, while the back translation also focused on ensuring the adequacy of the conceptual equivalence for the original tool and the forward translation process. For the panel of experts, the item-content validity index was ≥0.9 for all the questions to be included. The Korean version of the LCSHBS-K was tested by ten participants in a pilot study on the clarity of the contents, comprehensibility, appropriateness of the questionnaire form, time taken to complete the questionnaire, and whether any issues arose in completing the questionnaire. The results indicated that <5 min was required to respond to each question, and no participant had a problem understanding the questions, based on which the final Korean version of the tool was confirmed.

### 2.6. Data Collection

The data collection period was from 29 August 2022 to 1 September 2022. To prepare for data collection, the online survey provider was informed of the participant inclusion and exclusion criteria, and the recruitment ad and online survey URL were opened on the panels of the online survey provider. Data were collected through a web survey using panels. The collected data were processed by the online survey provider to remove personal information and encode only the items required for the study. Data from 204 participants were transferred to the researchers. The recruitment ad for the web survey included the study purpose, content, and procedures, and also explained the rights and non-disclosure agreement before the survey. Consent for voluntary participation was also obtained. A reward was provided to the participants for completing the survey according to the policy of the online survey provider.

### 2.7. Statistical Analysis

The collected data were analyzed using IBM SPSS Amos and SPSS WIN 26.0 version (IBM, NY, USA). Descriptive statistics, including numbers and percentages, means and standard deviations, and minimum and maximum values, were obtained for the participant characteristics and main variables. The reliability analysis involved Cronbach’s α for internal consistency and Pearson’s correlation coefficients to identify the correlations with the health belief scale for adults developed by Moon [17] for concurrent validity. When a tool has been developed on a theoretical basis with confirmed factor structures, it should be tested to determine whether the tool is valid in terms of population fluctuations. As a confirmatory factor analysis is more suitable than an explorative factor analysis [20], the former was used. The model fit for a tool is an absolute fit index (AFI) used to comparatively analyze the fitness of the model to the sample data. Thus, the model fit was tested based on CMIN (χ^2^/df) < 2 [21], SRMR < 0.08 [22], RMSEA < 0.06, GFI > 0.9 [23], and CFI > 0.9 [24] as a comparative fit index. The discriminant validity was tested based on AVE > r^2^ [25]. Using confirmatory factor analysis, the factor loading and standard errors were verified for each question per factor. To test the convergent validity and construct reliability, the average variance extracted (AVE) > 0.5 and composite reliability (CR) > 0.7 were calculated. The significance level was set at <0.05.

## 3. Results

### 3.1. Participant Characteristics

The mean age of all the participants was 55.49 years (SD = 5.07), and most of the participants were male (75.5%), married (75.5%), and had an education level above high school (75.5%). The number of individuals without religion and residing in an urban area was 116 (56.9%) and 185 (90.7%), respectively. The mean number of cohabiting family members was 2.89 (SD = 1.19). The mean economic status, perceived stress, and health status were 3.33 (SD = 0.93), 3.39 (SD = 0.75), and 2.90 (SD = 0.78), respectively. A total of 83 (40.7%) participants were cigarette-smokers, and 48 (23.5%) of the participants were liquid-type e-cigarette smokers. The average total period of cigarette smoking was 29.55 years (SD = 8.12), and the mean frequency of cigarette smoking was 12.18 cigarettes (SD = 7.77) per day. The total average period of e-cigarette smoking was 2.62 years (SD = 1.50), and the mean frequency of e-cigarette smoking was 8.62 times (SD = 4.48) per day (Table 1).

### 3.2. Construct Validity of LCSHBS-K

**Figure 1:**
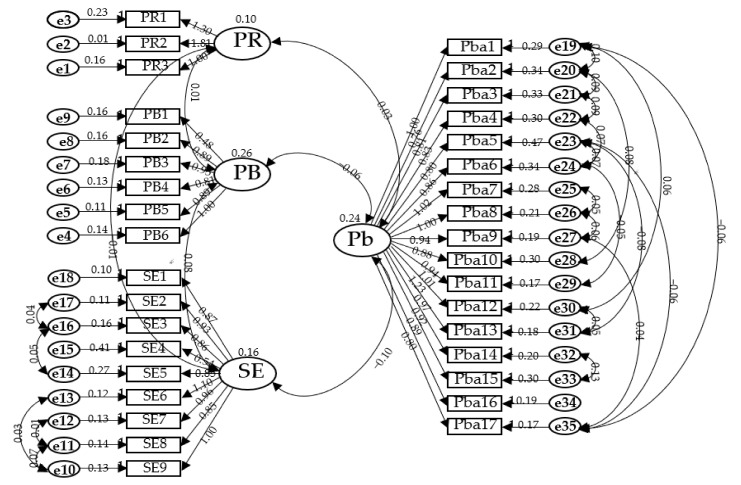
A modified and unstandardized model of LCSHBS-K. Notes: PR, perceived risk; PB, perceived benefit; Pba, perceived barrier; SE, self-efficacy.

The fit of the LCSHBS-K measurement model did not meet the criteria of the CMIN, SRMR, RMSEA, CFI, or GFI values. The modification indexes were verified to increase the fitness, and a modified model was produced (Figure 1). The goodness of fit of the revised model did not meet the GFI criteria (0.81 (criteria > 0.9)) but satisfied all the other criteria: CMIN = 1.69 (criteria < 2), SRMR = 0.06 (criteria < 0.08), RMSEA = 0.058 (criteria < 0.06), and CFI = 0.91 (criteria > 0.9). Therefore, we used the revised model as the final model (Table 2).

The mean values of the perceived risk, perceived benefit, perceived barrier, and self-efficacy were 6.44 ± 1.46, 18.73 ± 2.70, 36.51 ± 8.10, and 26.52 ± 3.54, respectively, and the factor loadings were ≥0.5 for most of the questions (Table 3).

When the dimensions of the LCSHBS-K were compared between the screened and the unscreened individuals, it was found that the perceived barrier was significantly higher in the unscreened than in the screened (t = −3.26, *p* = 0.001), and there was no difference in the other dimensions (Table 4).

### 3.3. Convergent and Discriminant Validity of LCSHBS-K

The correlation coefficient r among the scores in each dimension of the LCSHBS-K ranged from −0.51 to 0.38. The AVE values ranged from 0.45 to 0.60, and the perceived risk and perceived benefit were both above the criterion of 0.50 [26]. It satisfied the criterion of r^2^ < AVE of Fornell & Larcker [27], thus confirming the discriminant validity (Table 5).

### 3.4. Concurrent Validity and Reliability of LCSHBS-K

The perceived risk, perceived benefit, and self-efficacy in the dimensions of the LCSHBS-K and HBS showed significant positive correlations, but there was no significant correlation between the perceived barrier and HBS. The CR ranged between 0.81 and 0.93, above the criterion of 0.70 [26]. The reliability of the LCSHBS-K ranged from 0.78 to 0.93, a level of reliability similar to the dimensions of the original LCSHBS [12]. Thus, the internal consistency of the tool was verified (Table 6).

## 4. Discussion

This study translated and produced a Korean version of the LCSHBS in South Korea and verified the suitability of the LCSHBS-K by testing its validity and reliability in Korean adults at high risk for lung cancer. The LCSHBS has verified validity and reliability in English-speaking countries, where it was originally developed, as well as in China, which shares cultural similarities with South Korea [28]. In addition, the LCSHBS was translated into a Spanish version, and its validity and reliability were verified [29].

Verification of the validity of a tool is essential. The American Educational Research Association stated that the evidence for validity cannot be supported using a single approach [30]. Hence, the present study aimed to test the validity of the LCSHBS-K using various methods.

The present study complied with the guidelines for tool development to translate the LCSHBS based on the current status in South Korea. First, to ensure the cultural equivalence between the country where the original tool was developed and South Korea, where the tool is to be applied, the Brislin method was used to produce the Korean version of the tool. For this purpose, bilingual experts familiar with both cultures and a panel of experts, including lung cancer and nursing specialists, were recruited to ensure the content validity of the tool. Items that satisfied the item-content validity by the panel of experts were selected.

To ensure the construct validity of the LCSHBS-K, a revised version of the initial model was adopted, as it satisfied the CMIN, SRMR, RMSEA, and CFI criteria. The factor structures of the English and Korean versions of the LCSHBS were similar.

To ensure the concurrent validity of the LCSHBS-K, the correlation coefficients for the LCSHBS-K and the health belief scale for adults developed by Moon [17] were obtained and showed a significant correlation for the overall scale. However, the correlation coefficient for the perceived barrier was 0.08, indicating a lack of correlation, in contrast to all the other dimensions, which showed significant correlations. This is similar to a study of high-risk Chinese-American smokers who were interviewed about their beliefs regarding lung cancer screening using low-dose CT [31]. The responses of the participants on perceived severity, perceived benefits, and perceived susceptibility were similar, but the responses to the perceived barriers varied across the participants [31]. Lei et al. reported that the obstacles reported by participants regarding lung cancer screening included emotional factors, inadequate knowledge of lung cancer screening, economic factors, physical barriers, ill-advised beliefs regarding health maintenance, language barriers, and social stigma regarding screening tests, which varied widely across the participants [31]. Hence, the barriers preventing target behaviors in participants could be similar or vary widely. Similarly, in the LCSHBS, the perceived barrier also includes a higher number and variety of questions than in the other dimensions; therefore, the total score, as well as the content, should be examined for this particular dimension when using the LCSHBS. On the other hand, when health beliefs were compared between individuals who received lung cancer screening and those who did not, statistical differences were found only in the perceived barriers. The Spanish version of LCSHBS also showed no differences in all dimensions [29]. However, the difference in scores between the two groups in both the Korean and Spanish versions was consistent with the theoretical direction of the EHBM [29].

All the convergent and discriminant validity criteria for the LCSHBS-K were satisfied. For all the factors, the AVE and CR were ≥0.90 and ≥0.98, respectively. The r across the factors was −0.51 to 0.38, which satisfied r^2^ < AVE.

The internal consistency of the LCSHBS-K in this study showed a Cronbach’s α of 0.80. The reliability of dimensions was relatively high, ranging between 0.78 and 0.93, and was similar in the reliabilities of the English and Chinese versions. These results confirmed the internal consistency of the tool [12,28].

The results of this study demonstrate that the LCSHBS-K is a valid and reliable tool for assessing health beliefs regarding lung cancer screening. The LCSHBS-K will contribute to a more accurate assessment and in-depth understanding of the levels and types of health beliefs regarding lung cancer screening in Korean patients at high risk for lung cancer. This accurate understanding may lead to a positive change in these health beliefs, which would ultimately promote decision-making regarding lung cancer screening for positive health outcomes [9]. Therefore, the LCSHBS-K is anticipated to enable personalized interventions to develop various policies and programs aimed at increasing lung cancer screening in the Korean population, especially for those at high risk of lung cancer. Although the LCSHBS-K is available for measuring Korean speakers’ health beliefs regarding lung cancer screening, this study has several limitations. First, the characteristics of the participants recruited via online platforms may have influenced the results. Since subjects who did not use smartphones or PCs frequently or who did not know how to use them were excluded, the participants might have differences in age or knowledge level compared to those excluded from the study. Therefore, future study is required to include a variety of participants, including those excluded from this study. Second, since the Korean HBS compared to check concurrent validity—even though it has been used several times in Korea—was not a scale for lung cancer or other cancer screening, but a scale for measuring health beliefs for overall health promotion behaviors to prevent chronic disease, careful interpretation is required. Third, it was necessary to include in the model several correlations between the perceived barriers scale items to obtain a satisfactory model fit for a tool. These correlated residuals suggest that those items share some variance beyond the generic perceived barrier theme, indicating that this is a heterogeneous dimension that may comprise several subdimensions. Future research should explore putative subdimensions for perceived barriers in lung cancer screening.

## 5. Conclusions

This study was conducted to translate the English version of the LCSHBS to suit the Korean population, and to verify the suitability of the LCSHBS-K by testing its validity and reliability. The results confirmed that the LCSHBS-K was a valid and reliable tool to assess health beliefs regarding lung cancer screening in a Korean population at high risk of lung cancer. Although various factors affect lung cancer screening, it is clear that health beliefs also affect lung cancer screening status and intention. Thus, the LCSHBS-K may allow for a more accurate assessment and in-depth understanding of the levels and types of health beliefs regarding lung cancer screening in patients at high risk for lung cancer. It will also make it possible to prepare more detailed and personalized interventions to increase participation in lung cancer screening among high-risk groups of lung cancer.

## Figures and Tables

**Table 1:** Characteristics of the participants (n = 204).

Characteristics		N (%)	M ± SD	Range
Age (years)			55.49 ± 5.07	50–74
Sex	Male	154 (75.5)		
	Female	50 (24.5)		
Education	High school	50 (24.5)		
	College or University	122 (59.8)		
	>Master course	32 (15.7)		
Marital status	Yes	154 (75.5)		
	No	50 (24.5)		
Number of families living together			2.89 ± 1.19	1–6
Religion	Yes	88 (43.1)		
	No	116 (56.9)		
Residence	Urban	185 (90.7)		
	Rural	19 (9.3)		
Economic status			3.33 ± 0.93	1–5
Perceived stress			3.39 ± 0.75	1–5
Health status			2.90 ± 0.78	1–5
Screening test: CT	Yes	60 (29.4)		
	No	144 (70.6)		
Past history: pulmonary disease	Yes	20 (9.8)		
	No	184 (90.2)		
Cigarette smoking period (years)			29.55 ± 8.12	15–54
Cigarette smoking (number/day)			12.18 ± 7.77	0–60
E-cigarette (cigarette-type)	Yes	83 (40.7)		
	No	121 (59.3)		
E-cigarette (liquid type)	Yes	48 (23.5)		
	No	156 (76.5)		
E-cigarette smoking period (years)			2.62 ± 1.50	0–5
E-cigarette smoking (number/day)			8.62 ± 4.48	1–20

Notes: CT, computed tomography; M, mean; SD, standard deviation.

**Table 2:** Model fit of LCSHBS-K (n = 204).

Variable		LCSHBS-K			
	CMIN	SRMR	RMSEA	CFI	GFI
Measurement model	2.38	0.07	0.082	0.81	0.72
Modified model	1.69	0.06	0.058	0.91	0.81

Notes: LCSHBS-K, Lung Cancer Screening Health Belief Scale-Korean version; CMIN. Minimum chi-square; evaluation criterion of standardized CMIN (χ^2^/df) is <2, SRMR < 0.08, RMSEA < 0.06, CFI ≥ 0.9, GFI ≥ 0.9; SRMR, standardized root mean squared residual; RMSEA, root mean square error of approximation; CFI, comparative fit index; GFI, goodness of fit index.

**Table 3:** Factor loading of the LCSHBS-K (n = 204).

Items (ID)	M ± SD	Factor Loading
Perceived risk (F1)	6.44 ± 1.46	
1. It is likely that I will get lung cancer sometime in my lifetime. (PR1)	2.46 ± 0.64	0.65
2. It is likely that I will get lung cancer in the next ten years. (PR2)	2.11 ± 0.59	0.99
3. It is likely that I will get lung cancer in the next five years. (PR3)	1.87 ± 0.51	0.63
Perceived benefit (F2)	18.73 ± 2.70	
1. Having a lung scan will help find lung cancer early. (PB1)	3.24 ± 0.47	0.80
2. Having a lung scan will lower my chances of dying from lung cancer. (PB2)	3.10 ± 0.58	0.81
3. Having a lung scan will help me not to worry as much about lung cancer. (PB3)	3.03 ± 0.64	0.75
4. Having a lung scan will help me plan for the future. (PB4)	3.11 ± 0.55	0.75
5. Having a lung scan will help my family not worry as much. (PB5)	3.18 ± 0.56	0.73
6. Having a lung scan will give me peace of mind. (PB6)	3.07 ± 0.64	0.52
Perceived barrier (F3)	36.51 ± 8.10	
1. I might put off having a lung scan because I worry about finding something wrong. (Pba1)	2.33 ± 0.74	0.73
2. I might put off having a lung scan because I do not have the time. (Pba2)	2.28 ± 0.71	0.73
3. I might put off having a lung scan because I do not have a regular healthcare provider. (Pba3)	2.39 ± 0.70	0.69
4. I might put off having a lung scan because no one in my family had lung cancer. (Pba4)	2.33 ± 0.69	0.58
5. I might put off having a lung scan because the cost would be a problem. (Pba5)	2.55 ± 0.80	0.50
6. I might put off having a lung scan because I do not have any lung problems or symptoms. (Pba6)	2.50 ± 0.72	0.59
7. I might put off having a lung scan because transportation would be a problem. (Pba7)	2.05 ± 0.73	0.57
8. I might put off having a lung scan because I am afraid the lung scan will damage my lungs. (Pba8)	1.97 ± 0.67	0.54
9. I might put off having a lung scan because I have had a bad experience with a hospital or healthcare provider. (Pba9)	1.94 ± 0.64	0.67
10. I might put off having a lung scan because I do not know enough about the test. (Pba10)	2.36 ± 0.70	0.69
11. I might put off having a lung scan because I think I am too old to benefit from screening for lung cancer. (Pba11)	1.99 ± 0.62	0.71
12. I might put off having a lung scan because I am a smoker. (Pba12)	2.07 ± 0.68	0.64
13. I might put off having a lung scan because I would rather not know if I have any lung problems. (Pba13)	2.02 ± 0.74	0.72
14. I might put off having a lung scan because I worry about feeling like a social outcast for smoking. (Pba14)	1.98 ± 0.66	0.82
15. I might put off having a lung scan because I worry about being blamed for having smoked. (Pba15)	1.89 ± 0.71	0.73
16. I might put off having a lung scan because it is not worth the effort. (Pba16)	2.02 ± 0.62	0.74
17. I might put off having a lung scan because I do not trust the healthcare system. (Pba17)	1.83 ± 0.57	0.62
Self-efficacy (F4)	26.52 ± 3.54	
1. How confident are you that you can make an appointment to have a lung scan? (SE1)	2.94 ± 0.47	0.74
2. How confident are you that you can find the time to have a lung scan? (SE2)	3.01 ± 0.50	0.68
3. How confident are you that you can find transportation to get to and from the clinic/hospital to have a lung scan? (SE3)	3.07 ± 0.53	0.73
4. How confident are you that you can get enough information about having a lung scan? (SE4)	2.76 ± 0.68	0.79
5. How confident are you that you can cover the cost of a lung scan, if needed? (SE5)	2.90 ± 0.63	0.55
6. How confident are you that you can get a lung scan even if you are worried about the results? (SE6)	3.01 ± 0.57	0.33
7. How confident are you that you can have a lung scan even if you do not know what to expect about the procedure? (SE7)	2.88 ± 0.53	0.66
8. How confident are you that you can even if you are anxious about the process? (SE8)	2.96 ± 0.51	0.75
9. How confident are you that you can even if you are anxious about the results? (SE9)	2.98 ± 0.55	0.75

Notes: LCSHBS-K, Lung Cancer Screening Health Belief Scale-Korean version; M, mean; SD, standard deviation.

**Table 4:** Mean differences between screened and unscreened individuals of the LCSHBS-K (n = 204).

Variables	Range	Total (n = 204)	Screened (n = 60)	Unscreened (n = 144)	t (*p*)
		M ± SD			
Perceived risk	3~12	6.44 ± 1.45	6.65 ± 1.41	6.35 ± 1.47	1.32 (0.188)
Perceived benefit	6~24	18.73 ± 2.70	19.02 ± 2.69	18.61 ± 2.70	0.98 (0.329)
Perceived barrier	17~68	36.512 ± 8.10	33.72 ± 8.56	37.68 ± 7.64	−3.26 (0.001)
Self-efficacy	9~36	26.52 ± 3.54	27.22 ± 3.67	26.24 ± 3.45	1.81 (0.071)

Notes: LCSHBS-K, Lung Cancer Screening Health Belief Scale-Korean version; M, mean; SD, standard deviation.

**Table 5:** Convergent and discriminant validity of the LCSHBS-K (n = 204).

Variables	Perceived Risk	Perceived Benefit	Perceived Barrier	Self-Efficacy	AVE	r^2^ < AVE
	r (r^2^)					
Perceived risk	1				0.60	Satisfied
Perceived Benefit	0.02 (0.00)	1			0.54	Satisfied
Perceived Barrier	0.17 (0.03)	−0.26 (0.07)	1		0.45	Satisfied
Self-efficacy	−0.08 (0.01)	0.38 (0.15)	−0.51 (0.26)	1	0.46	Satisfied

Notes: LCSHBS-K, Lung Cancer Screening Health Belief Scale-Korean version; r, Pearson correlation coefficient; AVE, average variance extracted.

**Table 6:** Concurrent validity and reliability of the LCSHBS-K (n = 204).

Variables	Items	HBS	Cronbach’ a		CR
		r (*p*)	Korean Version	Original	
Perceived risk	3	0.23 (0.001)	0.78	0.88	0.81
Perceived benefit	6	0.28 (<0.001)	0.87	0.80	0.87
Perceived barrier	17	0.08 (0.241)	0.93	0.89	0.93
Self-efficacy	9	0.22 (0.002)	0.88	0.92	0.88

Notes: LCSHBS-K, Lung Cancer Screening Health Belief Scale-Korean version; r, Pearson correlation coefficient; HBS, health belief scale; CR, composite reliability.

## Data Availability

Data sharing is not applicable.

## References

[1] Carter-Harris L., Ceppa D.P., Hanna N., Rawl S.M. (2017). Lung cancer screening: What do long-term smokers know and believe?. Health Expect..

[2] Korean Statistical Information Service Statistical Results of Causes of Death in 2021. https://kostat.go.kr/board.es?mid=a10301010000&&bid=218&act=view&list_no=420715.

[3] Korean Statistical Information Service 5-Year Relative Survival Rate for 24 Cancer Types/Time of Cancer Occurrence/Gender in 2020. https://kosis.kr/statHtml/statHtml.do?orgId=117&tblId=DT_117N_A00021&vw_cd=MT_ZTITLE&list_id=F_35&scrId=&seqNo=&lang_mode=ko&obj_var_id=&itm_id=&conn_path=MT_ZTITLE&path=%252FstatisticsList%252FstatisticsListIndex.do.

[4] Lei F., Lee E. (2019). Barriers to lung cancer screening with low-dose computed tomography. Oncol. Nurs. Forum.

[5] National Lung Screening Trial Research Team (2011). Reduced lung-cancer mortality with low-dose computed tomographic screening. N. Engl. J. Med..

[6] International Early Lung Cancer Action Program Investigators (2006). Survival of patients with stage I lung cancer detected on CT screening. N. Engl. J. Med..

[7] Jang S.H., Sheen S., Kim H.Y., Yim H.W., Park B.Y., Kim J.W., Park I.K., Kim Y.W., Lee K.Y., Lee K.S. (2015). The Korean guideline for lung cancer screening. J. Korean Med. Assoc..

[8] Lu M.M., Zhang T., Zhao L.H., Chen G.M., Wei D.H., Zhang J.Q., Zhang X.P., Shen X.R., Chai J., Wang D.B. (2018). The relationship between demands for lung cancer screening and the constructs of health belief model: A cross-sectional survey in Hefei, China. Psychol. Health Med..

[9] Carter-Harris L., Davis L.L., Rawl S.M. (2016). Lung Cancer Screening Participation: Developing a Conceptual Model to Guide Research. Res. Theory Nurs. Pract..

[10] Rosenstock I.M. (1974). The Health Belief Model and Preventive Health Behavior. Health Educ. Monogr..

[11] Rosenstock I.M., Stretcher V.J., Becker M.H. (1988). Social learning theory and the Health Belief Model. Health Educ. Q..

[12] Carter-Harris L., Slaven J.E., Monohan P., Rawl S.M. (2017). Development and psychometric evaluation of the lung cancer screening health belief scales. Cancer Nurs..

[13] Wood D.E., Kazerooni E.A., Baum S.L., Eapen G.A., Ettinger D.S., Hou L., Jackman D.M., Klippenstein D., Kumar R., Lackner R.P. (2018). Lung Cancer Screening, Version 3.2018, NCCN Clinical Practice Guidelines in Oncology. J. Natl. Compr. Cancer Netw..

[14] Tak J.K. (2007). Psychological Testing: An Understanding of Development and Evaluation Method.

[15] Comrey A.L., Lee H.B. (1992). A First Course in Factor Analysis.

[16] Wolf E.J., Harrington K.M., Clark S.L., Miller M.W. (2013). Sample size requirements for structural equation models: An evaluation of power, bias, and solution propriety. Educ. Psychol. Meas..

[17] Moon J.S. (1990). A Study of Instrument Development for Health Belief of Korean Adults. Unpublished Doctoral Dissertation.

[18] Mokkink L.B., Terwee C.B., Patrick D.L., Alonso J., Stratford P.W., Knol D.L., Bouter L.M., de Vet H.C. (2010). The COSMIN checklist for assessing the methodological quality of studies on measurement properties of health status measurement instruments: An international Delphi study. Qual. Life Res..

[19] Brislin R.W., Lonner W.J., Berry J.W. (1986). The Wording and Translation of Research Instruments. Field Methods in Cross-Cultural Research.

[20] Harrington D. (1990). Confirmatory Factor Analysis.

[21] Byrne B.M. (2010). Structural Equation Modeling with AMOS: Basic Concepts, Applications, and Programming.

[22] Hu L.T., Bentler P.M. (1999). Cutoff criteria for fit indexes in covariance structure analysis: Conventional criteria versus new alternatives. Struct. Equ. Model..

[23] Hooper D., Coughlan J., Mullen M. (2008). Structural equation modeling: Guidelines for determining model fit. Electron J. Bus. Res. Methods.

[24] Jöreskog K.G., Sörbom D. (1982). Recent developments in structural equation modeling. J. Mark. Res..

[25] Bentler P.M. (1990). Comparative Fit Indexes in Structural Models. Psychol. Bull..

[26] Anderson J.C., Gerbing D.W. (1981). Structural equation modeling in practice: A review and recommended two-step approach. Psychol. Bull..

[27] Fornell C., Larcker D.F. (1981). Evaluating structural equation models with unobservable variables and measurement error. J. Mark. Res..

[28] Lin Y.A., Carter-Harris L., Yang J.N., Lin X.J., Huang F.F. (2022). Adaptation and validation of the Chinese version of the lung cancer screening health belief scales. BMC Public Health.

[29] Carter-Bawa L., Schofield E., Atkinson T.M., Ostroff J.S. (2022). Development and psychometric evaluation of the Spanish version of the lung cancer screening health belief scale. Eur. J. Cancer Care.

[30] American Educational Research Association [AERA], American Psychological Association [APA], National Council on Measurement in Education [NCME] (2014). Standards for Educational and Psychological Testing.

[31] Lei F., Chen W.T., Brecht M.L., Zhang Z.F., Lee E. (2022). Health beliefs toward lung cancer screening among Chinese American high-risk smokers: Interviews based on Health Belief Model. Int. J. Nurs. Sci..

